# Recapitulating in vivo-like plasticity of glioma cell invasion along blood vessels and in astrocyte-rich stroma

**DOI:** 10.1007/s00418-017-1604-2

**Published:** 2017-08-19

**Authors:** Pavlo Gritsenko, William Leenders, Peter Friedl

**Affiliations:** 1 Microscopical Imaging of the Cell, Department of Cell Biology, Radboud Institute for Molecular Life Sciences, Radboud University Medical Center, Nijmegen, The Netherlands; 2 Department of Pathology, Radboud University Medical Center, Nijmegen, The Netherlands; 3 Department of Genitourinary Medical Oncology, David H Koch Center for Applied Research of Genitourinary Cancers, The University of Texas, MD Anderson Cancer Center, Houston, TX USA; 4 Cancer Genomics Centre (CGC.nl), 3584 Utrecht, The Netherlands

**Keywords:** Glioma invasion, Organotypic culture, Perivascular invasion, Astrocyte scaffolds, Multicellular networks

## Abstract

Diffuse invasion of glioma cells into the brain parenchyma leads to nonresectable brain tumors and poor prognosis of glioma disease. In vivo, glioma cells can adopt a range of invasion strategies and routes, by moving as single cells, collective strands and multicellular networks along perivascular, perineuronal and interstitial guidance cues. Current in vitro assays to probe glioma cell invasion, however, are limited in recapitulating the modes and adaptability of glioma invasion observed in brain parenchyma, including collective behaviours. To mimic in vivo-like glioma cell invasion in vitro, we here applied three tissue-inspired 3D environments combining multicellular glioma spheroids and reconstituted microanatomic features of vascular and interstitial brain structures. Radial migration from multicellular glioma spheroids of human cell lines and patient-derived xenograft cells was monitored using (1) reconstituted basement membrane/hyaluronan interfaces representing the space along brain vessels; (2) 3D scaffolds generated by multi-layered mouse astrocytes to reflect brain interstitium; and (3) freshly isolated mouse brain slice culture ex vivo. The invasion patterns in vitro were validated using histological analysis of brain sections from glioblastoma patients and glioma xenografts infiltrating the mouse brain. Each 3D assay recapitulated distinct aspects of major glioma invasion patterns identified in mouse xenografts and patient brain samples, including individually migrating cells, collective strands extending along blood vessels, and multicellular networks of interconnected glioma cells infiltrating the neuropil. In conjunction, these organotypic assays enable a range of invasion modes used by glioma cells and will be applicable for mechanistic analysis and targeting of glioma cell dissemination.

## Introduction

Gliomas represent the most common primary brain tumor type in adults, with glioblastoma as one of the most detrimental cancers in humans (Wen and Reardon 2016). The high lethality of glioma patients is mainly caused by diffuse invasion of glioma cells into the brain parenchyma, the extent of which typically precludes curative surgical resection and radiotherapy (Cuddapah et al. 2014). This diffuse invasive character is a relatively unique characteristic of gliomas and rarely seen in other brain cancers (Lenting et al. 2017). The structures of the brain tissue along which glioma cells migrate are complex and their microanatomic and molecular organization varies. Guiding structures include myelinated axons and astrocyte processes (white matter tracks) as well as basement membranes of blood vessels and the meninges (perivascular tracks) (Gritsenko et al. 2012; Cuddapah et al. 2014). Both brain regions contain hyaluronan as main component of the brain extracellular matrix (ECM) (Zimmermann and Dours-Zimmermann 2008). Disseminating glioma cells orient preferentially along aligned myelinated fibers and astrocyte processes throughout the white matter (Gritsenko et al. 2012; Cuddapah et al. 2014). In parallel, glioma cell invasion occurs along the interface between blood vessels and brain parenchyma (Farin et al. 2006; Gritsenko et al. 2012; Cuddapah et al. 2014). Glioma cells invade the brain tissue either individually, after detaching from neighboring glioma cells, or as cohesive groups preferentially moving along blood vessels (Farin et al. 2006; Winkler et al. 2009; Assanah et al. 2009; Hirata et al. 2012; Watkins et al. 2014; Baker et al. 2014; Krusche et al. 2016). In addition, glioma cells may form multicellular networks with long filaments connecting glioma cells in vivo, and these networks were recently implicated in glioma cell invasion and resistance signaling (Osswald et al. 2015).

In recent years, different assays were developed to model glioma microenvironments of the brain tissue and test the extent and mechanisms of glioma cell invasion (Rao et al. 2014; Rape et al. 2014). Widely used 2D assays are based on coating of the culture dish with ECM molecules, including laminin, fibronectin or collagen (Nakada et al. 2013; Chen and Nalbantoglu 2014). However, these models lack crucial parameters of 3D brain environments which modulate migration mechanisms, including (1) low substrate stiffness, (2) anatomically complex 3D organization, (3) 3D space confinements which enable adhesion-dependent and adhesion-independent migration, and (4) molecularly rich ECM composition maintained by brain cells and containing chemotactic factors (Rape et al. 2014). Reconstituted 3D migration assays are based on natural or synthetic hydrogels with various molecular components, including fibrillar collagen, reconstituted basement membrane (rBM) rich in laminin and type IV collagen, and composite hydrogels containing polyacrylamide, fibronectin and/or cross-linked hyaluronan (Gordon et al. 2003; Ulrich et al. 2009; Yang et al. 2010; Ananthanarayanan et al. 2011). In unperturbed brain, fibrillar collagens are expressed mainly along blood vessels but not in the parenchyma (Bellail et al. 2004; Gritsenko et al. 2012), and upregulated in a subset of clinical gliomas in the tumor mass and perivascular regions (Huijbers et al. 2010; Payne and Huang 2013; Motegi et al. 2014). Type I collagen scaffolds are effectively invaded by glioma cells (Kaufman et al. 2005; Yang et al. 2010; Frolov et al. 2016); however, the relevance of collagen as substrate for diffuse glioma cell infiltration beyond the tumor core remains unclear (Rape et al. 2014). rBM and cross-linked hyaluronan both represent key components of the brain stroma and, like synthetic hydrogels, provide soft environments similar to brain tissue; however, they lack other adhesion ligands and cell-derived brain structures, such as astrocyte networks and myelinated axons (Gritsenko et al. 2012).

Migration assays comprising brain-derived cells in monolayer culture, including primary astrocytes, provide 2D interaction scaffolds for glioma cells (Oliveira et al. 2005; Rath et al. 2013; Hong et al. 2015). Astrocyte monolayers support glioma cell invasiveness via gap-junction communications and by secretion of promigratory molecules (Oliveira et al. 2005; Rath et al. 2013; Hong et al. 2015). As 3D modification, primary rat astrocytes were combined with electrospun nanofiber scaffolds, and this approach revealed a contribution of astrocytes to single cell migration of glioma cells by secreting migration-enhancing  factors (Rao et al. 2014). However, both astrocyte monolayers and electrospun scaffolds lack the complexity and, likely, softness of 3D brain stroma (Rape et al. 2014).

Consequently, live brain slice assays are considered as “gold standard” recapitulating the complexity of brain tissue; however, they support mainly perivascular but not parenchymatous routes of glioma cell invasion (Alfi et al. 2002; Miao et al. 2015; Fayzullin et al. 2016). To this end, we hypothesize that the complexity of glioma invasion requires the combined application of a set of complementary in vitro models to enable an adaptive range of glioma invasion types, including collective perivascular invasion and network-like interstitial invasion patterns (Osswald et al. 2015, 2016).

We here developed a set of 3D assays mimicking brain-like structures to analyse glioma cell invasion patterns from 3D multicellular spheroids. When compared to in vivo invasion of the same cell types in the mouse brain and to histopathology of human lesions, each assay delivers dedicated in vivo-like invasion programs, including single cell migration, collective sheets and strands, and/or multicellular glioma networks.

## Materials and methods

### Antibodies and reagents

The following antibodies were used: anti-mouse β-catenin (mouse clone 14/beta-catenin, 1:100, BD Biosciences); anti-human β-catenin (rabbit polyclonal, 1:1000, Abcam); anti-mouse N-cadherin (mouse, clone GC-4, 1:200, Sigma); anti-mouse laminin (rabbit polyclonal, 1:100, Sigma); anti-human collagen-IV (mouse, clone Col-94, 1:300, Sigma); anti-human vimentin (rabbit, SP20 clone, human specific, 1:300, Thermo Scientific), anti-bovine glial fibrillary acidic protein (GFAP) (chicken polyclonal, 1:1000, Abcam); anti-human nestin (rabbit polyclonal, human specific, 1:100, Millipore). Primary antibodies were visualized with secondary AlexaFluor-conjugated goat-anti-mouse, goat-anti-rabbit or goat-anti-chicken polyclonal antibodies (Invitrogen; 5 µg/ml). For background controls, isotype-matched unspecific mouse IgG (BD Biosciences) was used for monoclonal antibody stainings, and for polyclonal antibody stainings samples were incubated only with secondary AlexaFluor-conjugated antibodies. Cell nuclei were stained with 4′,6-diamidino-2-phenylindole (DAPI; 2.5 µg/ml). F-actin was labelled with AlexaFluor-conjugated phalloidin (Invitrogen). Growth factor-reduced reconstituted basement membrane (rBM) (Matrigel, BD Biosciences; 9.8 mg/ml) was used for rBM interface culture.

### Cell lines and culture

Human glioblastoma E-98 and E-468 cells were maintained as patient-derived xenografts by serial subcutaneous (E-98) and intracerebral (E-468) inoculation without in vitro culture (Claes et al. 2008). E-468 cells were freshly isolated from mouse brain 7–8 days prior to each spheroid preparation for migration assays to minimize adaptation to in vitro culture. E-468 cells were maintained in neurobasal medium (Invitrogen) supplemented with human EGF (20 ng/ml), human bFGF (20 ng/ml), B27 Supplement (1:50), l-glutamine (2 mM) (all from Invitrogen), heparin (2 μg/ml, Sigma), penicillin (100 U/ml) and streptomycin (100 μg/ml; both PAA). Cells were maintained for up to two passages (E-468) in 2D culture on flasks coated with growth factor-reduced rBM (BD Biosciences; 30 µg/ml in PBS). Accutase digestion (10 min, 400–600 units/ml; Sigma) was used for cell detachment and dissociation of multicellular spheroids. A subline of E-98 cells was propagated in vitro in flasks for up to passage 35. Human glioblastoma U-251MG cells (kind gift from Dr. J. Schalkwijk, Nijmegen) were maintained permanently in in vitro culture. Primary mouse astrocytes immortalized with SV40 large T-antigen and additionally transformed with retrovirus pBabe puro H-Ras V12 (kindly provided by Amparo Acker-Palmer, Institute of Cell Biology and Neuroscience and BMLS, Goethe University Frankfurt, Germany) were maintained as described (Sawamiphak et al. 2010; Depner et al. 2016). H2B/eGFP-expressing U-251 and E-98 cells were generated by lentiviral transduction with pLenti6.2/V5-DEST™ Gateway (Invitrogen) containing histone2B/eGFP. For in vitro invasion assays murine astrocytes and human E-98 and U-251 cells were maintained in Dulbecco’s Modified Eagle’s Medium (DMEM; Invitrogen) supplemented with 10% fetal bovine serum (Sigma-Aldrich), penicillin (100 U/ml) and streptomycin (100 μg/ml; both PAA), l-glutamine (2 mM, Invitrogen) and sodium pyruvate (1 mM, Invitrogen).

### Generation of glioma cell spheroids

Glioma cell spheroids were generated using the hanging drop method (Korff and Augustin 1998). Cells were cultured in DMEM until subconfluency, detached with 1 mM EDTA/0.075% trypsin or with Accutase (400–600 units/ml; Sigma), washed with PBS, and maintained for 24 h in complete DMEM/methylcellulose (2.4%; Sigma) as hanging droplets (25 μL) containing 1000 (U-251, E-468) or 2000 (E-98) cells.

### Reconstituted basement membrane interface migration assays

3D rBM/hyaluronan interface cultures were generated by polymerizing growth factor-reduced rBM on a culture dish (30 min, 37 °C), followed by addition of glioma spheroids over polymerized rBM in complete DMEM with further incubation (2 h, 37 °C, 10% CO_2_), replacement of media by complete DMEM supplemented with sodium hyaluronate (Sigma, Cat: 53747, from Streptococcus equi, Mr ~ 1.5 – 1.8 × 10^6^ Da, 10 mg/ml) or methylcellulose (Sigma, Cat: M6385, viscosity 25 cP, 15 mg/ml) and incubation for 24–48 h. For rBM-plastic interface cultures, glioma spheroids were placed on 96-well plates (Greiner Bio One, PS, F-bottom, μClear, Black, CELLSTAR) coated with growth factor reduced rBM (30 µg/ml, diluted in PBS, preadsorbed overnight at 4 °C). After spheroid addition, cultures were incubated in complete DMEM or neurobasal media (2 h, 37 °C, 10% CO_2_), overlaid with growth factor reduced rBM (5 mg/ml, diluted in PBS) and incubated to allow rBM polymerization (30 min, 37 °C). After 24 or 48 h of migration culture in complete DMEM or neurobasal media, samples were fixed (4% PFA, 30 min, RT) and analysed by bright-field or confocal microscopy.

### 3D astrocyte scaffold invasion assay

3D astrocyte-derived scaffolds were generated by immortalized murine astrocytes maintained at high cell density in 96-well plates (Greiner Bio One, PS, F-bottom, μClear, Black, CELLSTAR; 20,000 cells/well, coated with growth factor-reduced rBM) for 2–3 days resulting in consolidated 3D scaffolds of up to 3–4 cell layers in height. Glioma cell spheroids expressing H2B/eGFP were cultured on top of astrocyte scaffolds (4–5 spheroids/well) for 2 days, fixed (4% PFA, 30 min, RT), and stained to visualize glioma cells (human specific anti-vimentin antibody), astrocytes and ECM proteins.

### Organotypic mouse brain slice invasion assay

To probe glioma cell invasion into 3D brain slices, the assay from (Montana and Sontheimer 2011) was modified for multicellular spheroid culture. Brains from female mice (5–6 weeks old; Charles River or Jackson Laboratories) were dissected, using the strain C57BL/6-Tg(TcraTcrb)1100Mjb/Crl (OT1) which was crossed with B6.Cg-Tg(CAG-DsRed*MST)1Nagy/J (dsRed) in our laboratory. Brains were freshly sectioned as 400 µm-thick tissue slices using a vibratome (Leica, VT1000 s). Slices were maintained on transwell insert membranes (Costar, 12-well plate; 8 µm pore diameter) in complete DMEM (37 °C, 5% CO_2_) for 1 h. Glioma cell spheroids expressing H2B/eGFP were added on top of brain slices (8–10 spheroids per slice), cultured in complete DMEM for 48 h, fixed (4% PFA, 1 h, 20 °C), washed and stained with human-specific anti-vimentin antibody to discriminate glioma cells from DsRed-expressing blood vessels.

### Confocal microscopy and quantification of glioma cell migration

Confocal microscopy (Olympus FV1000) was performed using long working distance 20 × NA 0.50 and 40 × NA 0.80 objectives at a vertical step size of 2–3 µm. 3D reconstruction of Z-stacks was performed using Imaris V.6.1.5 (Bitplane).

For quantitative image analysis, operator-assisted image segmentation of bright-field or confocal 3D stacks was performed [Fiji software, V.1.49 g (Schindelin et al. 2012)]. The average cell migration distance representing radial migration of glioma cells from spheroids was calculated according to the following formula:

$${\text{Average distance of cell migration}} = \sqrt {{{\text{Total cell area}} \mathord{\left/{\vphantom {{\text{Total cell area}}\pi }} \right. \kern-0pt} \pi }} - \sqrt {{{\text{Spheroid area}} \mathord{\left/{\vphantom {{\text{Spheroid area}}\pi }} \right. \kern-0pt} \pi }} .$$

### Glioblastoma xenografts in mouse brain

Female athymic Balb/C nu/nu mice (6–8 weeks old) were obtained from Charles River Laboratories and maintained under specific pathogen-free conditions at the central animal facility of Radboudumc, Nijmegen. The animal experiments were approved by the Ethical Committee on Animal Experiments of the Radboud University, Nijmegen, The Netherlands (RU-DEC-2013-251) and performed in accordance with the Dutch Animal Experimentation Act and the European FELASA protocol (www.felasa.eu/guidelines.php). U-251-Fluc-mCherry and E-98-Fluc-mCherry parental cells (Wurdinger et al. 2008; Mir et al. 2010) were cultured as spheroids in neurobasal media for 1 month, enzymatically dissociated by Accutase digestion, and intracranially implanted (5 × 10^5^ cells in 20 µL PBS) by guided injection into the right parieto-occipital hemisphere of isoflurane-anesthetized mice 2 mm from the midline.

### 3D reconstruction of glioma lesions

Paraffinized clinical samples from four anonymized glioma patients (primary glioblastoma) were obtained from the archives of the Department of Pathology, Radboudumc, Nijmegen. Informed patient consent and ethical committee approval for the use of (archival) brain tissue was obtained and the material was used in a manner compliant with the Declaration of Helsinki. Slices of 100 µm thickness were obtained by microtome slicing (HM 340E, Thermo Scientific Microm), deparaffinized (100% xylene), gradually rehydrated (sequential 100, 96, 70, 50% v/v ethanol/water), heated for antigen retrieval (98 °C, 15 min in Tris–EDTA, pH 9.0), incubated with blocking solution (0.1% Tween-20 and 1% bovine serum albumin in PBS) and stained with antibodies. To reach saturated antibody conditions and efficient washing in the 3D sample, prolonged incubation periods with primary and secondary antibodies and each washing step (0.1% tween-20/PBS; 0.05% NaN3) were 8–24 h at room temperature to ascertain antigen saturation and complete removal of unbound antibody. The presence of nestin in the absence of astrocytic and neuronal markers GFAP and myelin basic protein (MBP), respectively, was used to identify glioma cells (Kitai et al. 2010). 200 µm thick sections from glioblastoma xenografts (U-251, E-98 and E-468 cells) in mouse brains were obtained after fixation (4% PFA, 20 h) by vibratome slicing (Leica, VT1000 s) followed by counterstaining to detect murine astrocytes by anti-GFAP pAbs, basement membranes by anti-laminin pAb and glioma cells by human-specific anti-vimentin or anti-nestin pAbs.

## Results and discussion

### Glioma cell migration along reconstituted basement membrane interfaces

**Fig. 1:**
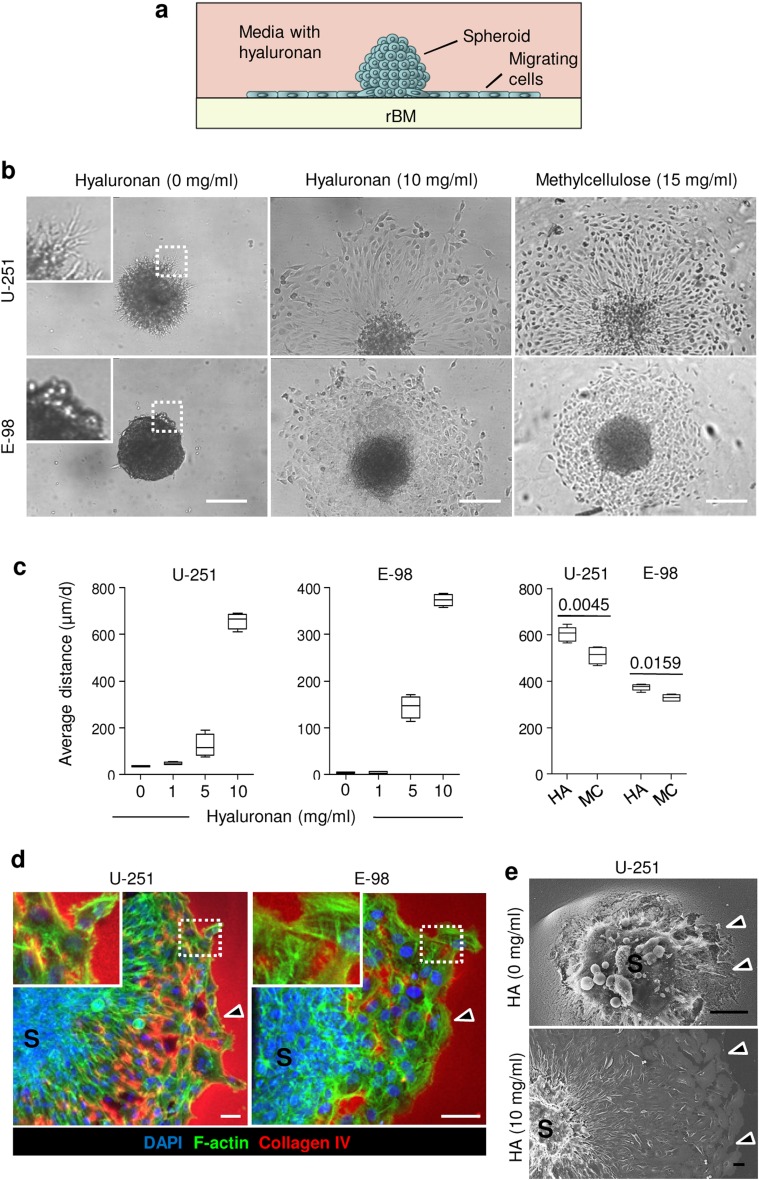
Reconstituted basement membrane/hyaluronan interface migration assay. **a** Assay design. **b** Radial migration of U-251 and E-98 cells from spheroids along the rBM–hyaluronan (HA) or methylcellulose (MC) interface after 1 day of culture, detected by bright-field microscopy. **c** Average distance migrated by U-251 and E-98 cells along the rBM/HA or rBM/MC interface at different concentration of HA or MC; values display medians (*black line*), 25/75 percentiles (*boxes*) and maximum/minimum (*whiskers*) from three independent experiments. *p* values, Mann–Whitney test. **d** 3D projection from confocal z-stack of U-251 and E-98 cell migration from multicellular spheroids (S) along rBM/HA interface (10 mg/ml HA concentration). *Arrowheads* indicate the invasion front. **e** Scanning electron microscopy of U-251 cells after 1 day of radial migration from spheroids (S) on rBM in media without or with HA (10 mg/ml). *Arrowheads*, invasion front with signs of degradation of rBM (HA, 0 mg/ml) or without rBM degradation (HA, 10 mg/ml). *Scale bars* 200 μm (**b**), 50 μm (**d**, **e**)

**Fig. 2:**
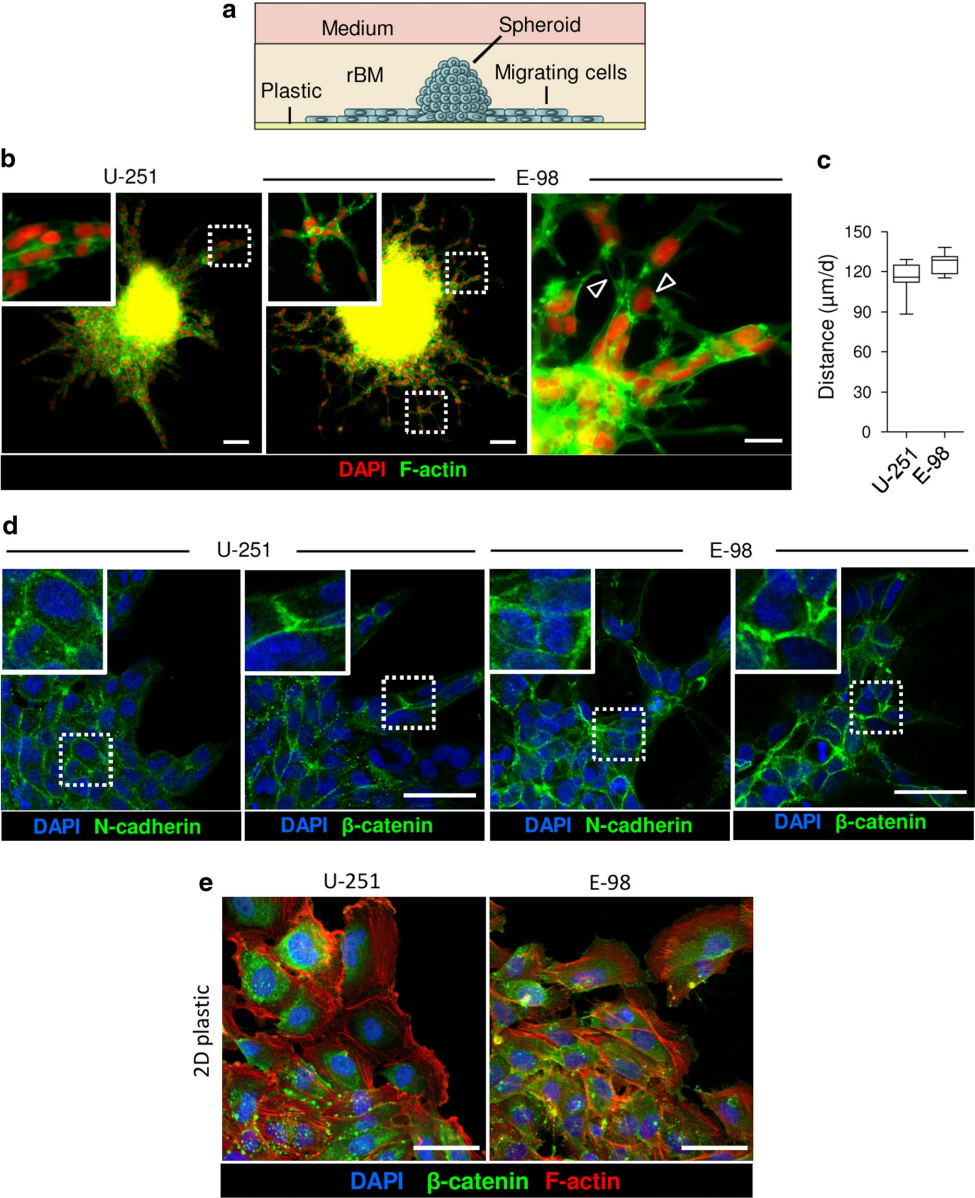
rBM-plastic interface migration assay. **a** Assay design. **b** Overviews of U-251 and E-98 cells after 2 days of radial migration from spheroids under rBM in neurobasal media. *Arrowheads*, focal cell–cell interactions. **c** Average migration distance of U-251 and E-98 cells under rBM. Values display median (*black line*), 25/75 percentiles (*boxes*) and maximum/minimum (*whiskers*) from three independent experiments. **d** Molecular topology of adherens junction proteins in U-251 and E-98 cells migrating under rBM. Images were obtained by epifluorescence (**b**) and confocal microscopy (**d**). **e** Maximum z-projection of U-251 and E-98 cells after 2 days of emigration from multicellular spheroids maintained on polystyrene surface coated with rBM. *Scale bars* 100 μm (**b**), 20 μm (zoomed insert **b**), 50 μm (**d**,**e**)

To recapitulate perivascular glioma cell invasion along interfaces formed by brain parenchyma and basement membranes, we used polymerized rBM, which comprises structural glycoproteins constituting basement membrane (Albini et al. 1987; Hughes et al. 2010), overlaid with hyaluronan, the most abundant component of interstitial brain ECM (Zimmermann and Dours-Zimmermann 2008) (Fig. 1a). When overlaid on 3D rBM without hyaluronan in the supernatant, U-251 glioma cells invaded into, but not along the rBM, and E-98 cells failed to establish radial migration but grew as compact spheroids (Fig. 1b). When hyaluronan was overlaid, both U-251 and E-98 cells developed sheet-like migration (Fig. 1b) with the speed increasing in dependence of the hyaluronan concentration reaching up to 10 mg/ml, a supra-physiological concentration at which hyaluronan formed a viscous, semi-solid solution (Fig. 1c, d). In gliomas, extracellular hyaluronan concentrations may range from 0.2 up to 5 mg/ml (Delpech et al. 1993; Sykova and Nicholson 2008). In control experiments using methylcellulose as non-physiological, inert polysaccharide in combination with rBM, migration of U-251 and E-98 cells was equally well supported (Fig. 1b, c). This indicates a generic pro-migratory function of a protein interface adjacent to viscous polysaccharide. In previous work, glioma cell migration was primarily assessed in single-cell migration assays, using cell suspensions after enzymatic dispersion (Nakada et al. 2013; Chen and Nalbantoglu 2014). However, when tested as tumor-like multicellular spheroids, which allow cells to establish cell–cell junctions, both U-251 and E-98 cells migrated collectively, as a cohesive sheet of cells, along the rBM-hyaluronan interface (Fig. 1d, e). Similarly, when spheroids were positioned at the interface between rBM overlaying the plastic substrate of the culture plate (Fig. 2a), U-251 glioma cells migrated as epithelial-like sheets and collective strands, whereas E-98 cells formed thinner strands and complex-shaped multicellular networks with cells retaining both linear junctions and connecting filaments between cell bodies (Fig. 2b, c). Glioma invasion modes observed in hyaluronan-rBM vs rBM-plastic interfaces may result from the different molecular and mechanical characteristics of the interface along which they migrated, including coverage of rBM proteins by hyaluronan which may provide confinement and further modulate ligand availability for cell adhesion systems and the stiffness of migration substrate. These parameters may cooperatively influence the retention of cell–cell junctions, migration mode and speed (Haeger et al. 2014; Canver et al. 2016; Asano et al. 2017; Bangasser et al. 2017). Accordingly, both glioma cell lines moving collectively under rBM established linear or focal adherens junctions at cell–cell contacts which were positive for N-cadherin and β-catenin, whereas glioma cells migrating on a 2D surface lacked junctional β-catenin and showed its redistribution to the cytoplasm (Fig. 2d, e). The average distance of glioma cell migration under rBM was decreased by 60–80%, compared to migration along rBM-hyaluronan interfaces (compare Fig. 2c with Fig. 1c), indicating speed regulation in dependence of variations of confinement and/or substrate stiffness.

### Invasion into 3D astrocyte scaffolds

**Fig. 3:**
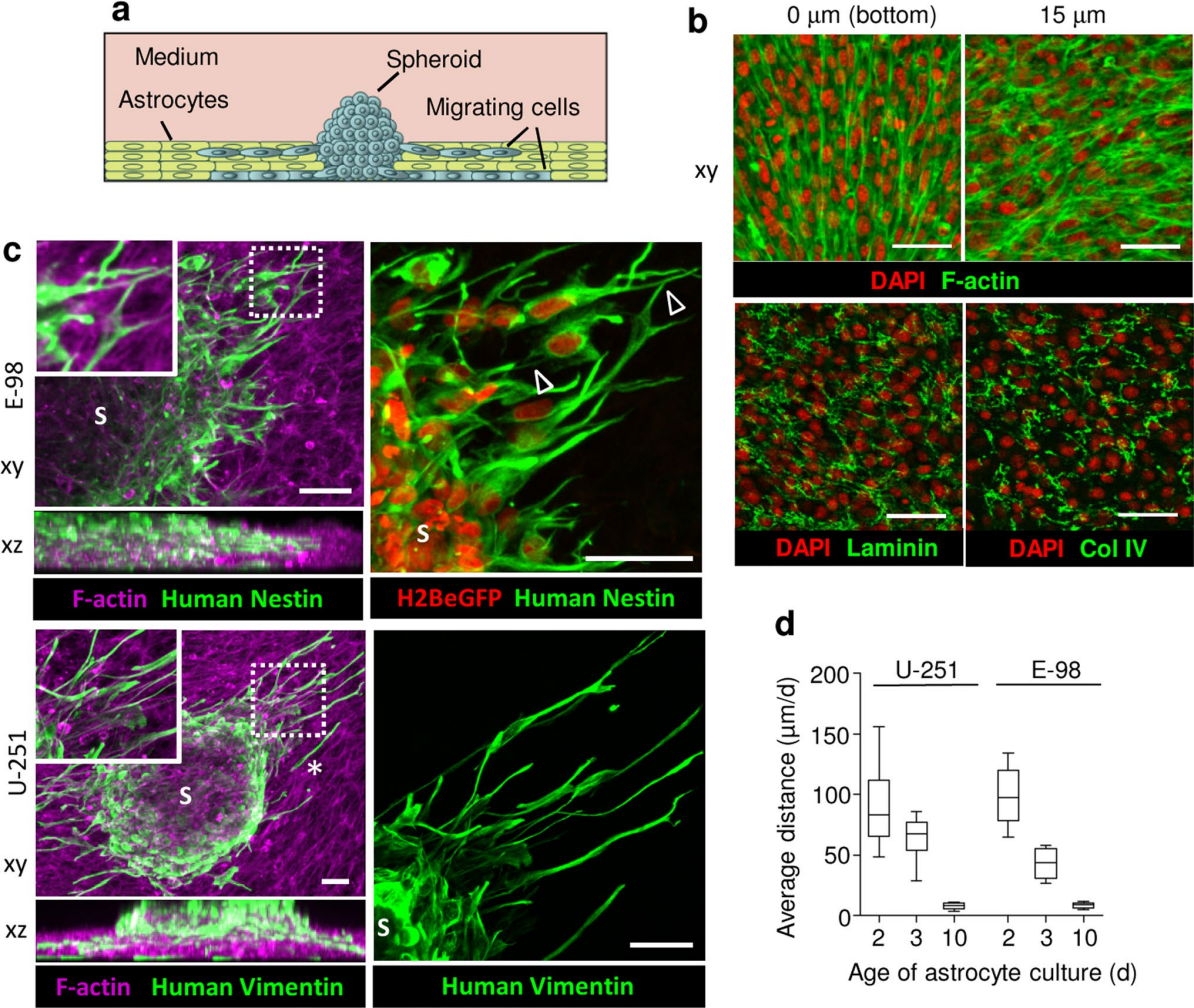
3D astrocyte scaffold invasion assay. **a** Assay design. **b** Confocal xy-sections of astrocyte culture (3 days) stained for F-actin, laminin and collagen type IV (Col IV). **c** 3D reconstruction (confocal z-stack, 90 μm, horizontal and orthogonal projections) of E-98 and U-251 cell invasion from spheroids (S) into 3-day old mouse astrocyte scaffolds. Glioma cells were identified by vimentin staining with human-specific antibody and constitutive expression of H2BeGFP in the nucleus, and murine astrocytes using phalloidin (F-actin). *Arrowheads* point to contacts between glioma cells via dendrite-like filaments. *Asterisk*, detached single cell. **d** Average migration distance of U-251 and E-98 cells invading astrocyte scaffolds matured for 2, 3 or 10 days before addition of glioma spheroids. Values display median (*black line*), 25/75 percentiles (*boxes*) and maximum/minimum (*whiskers*) from three independent experiments. *Scale bars* 50 μm

To reproduce diffuse glioma cell invasion in astrocyte-rich brain stroma we generated 3D scaffolds formed by immortalized murine astrocytes in hyperconfluent culture (Fig. 3a). Astrocytes proliferated and formed dense multicellular networks with up to three cell layers in thickness (~35 µm) during 3 days of culture (Fig. 3b). Astrocytes of the bottom layer typically aligned in parallel, whereas the top layer developed more varied and randomly orientated network-like organization (Fig. 3b). Hyperconfluent astrocyte cultures produced extracellular matrix molecules along their cell boundaries, including laminin and collagen IV (Fig. 3b), resulting in a dense cell- and ECM-rich 3D scaffold.

Glioma cells readily invaded astrocyte scaffolds, by aligning along and intercalating between astrocytes and penetrating all scaffold layers (Fig. 3c). The speed of glioma cell invasion correlated inversely with the duration of astrocyte scaffold conditioning, with average distances covered decreasing from ~100 µm/day in 2-day old scaffolds to less than 10 µm/day in 10-day old scaffolds (Fig. 3d). Notably, and in contrast to rBM based culture, U-251 and E-98 cells invaded astrocyte scaffolds as both, single cells (Fig. 3c, *asterisk*) and multicellular networks of individual cells connecting with neighbor cells via long dendrite-like filaments (Fig. 3c, arrowheads).

### Invasion into mouse brain slices

**Fig. 4:**
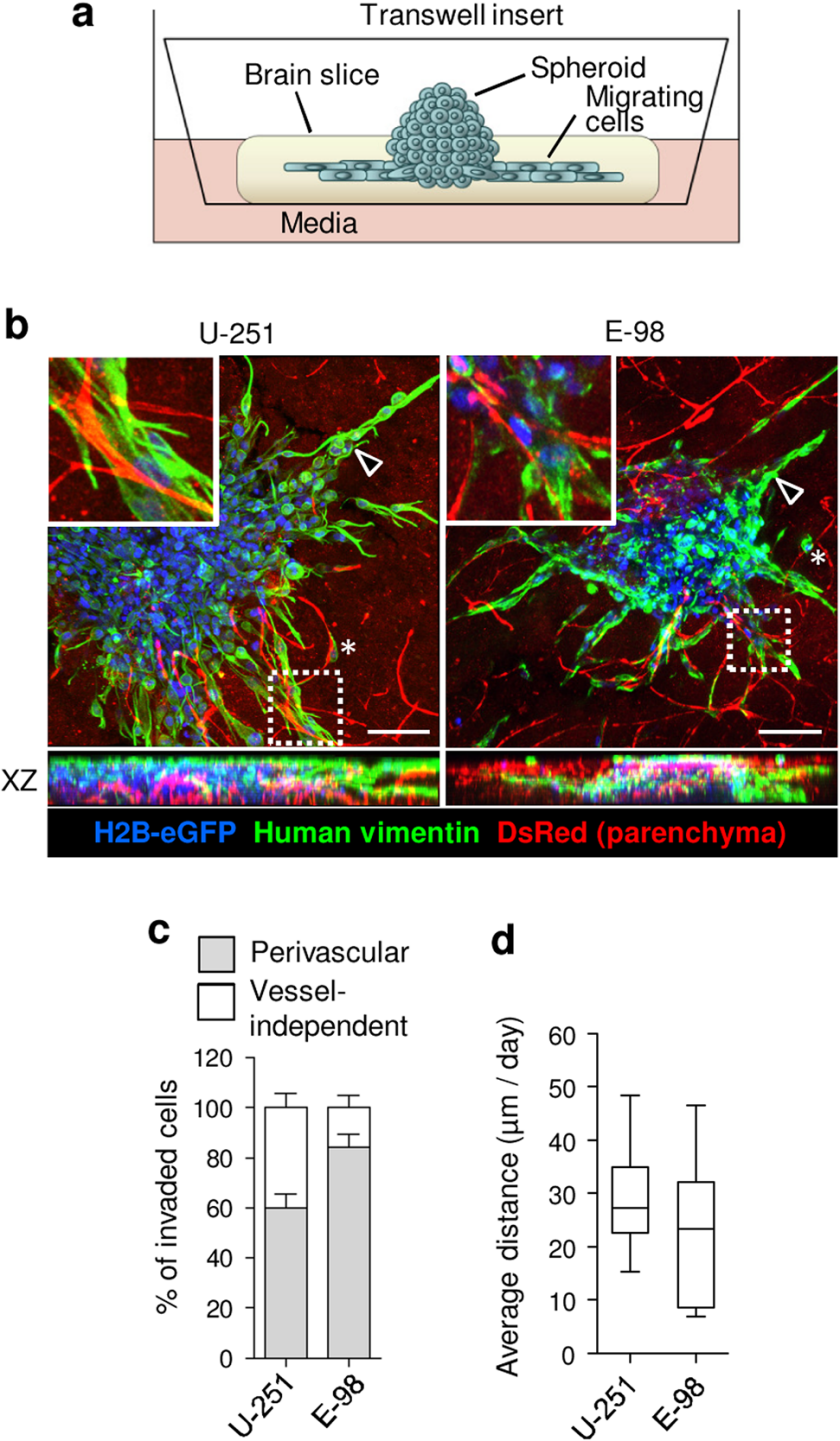
Organotypic mouse brain slice invasion assay. **a** Assay design. **b** 3D reconstruction (confocal z-stack, 90 μm, horizontal and orthogonal projections) of U-251 and E-98 cell migration from spheroids in mouse brain slices after 2 days of culture. *Arrowheads* indicate multicellular strands. *Asterisks*, detached single cells. Red signal originates from the DsRed mouse background, as contrast of vessels (bright signals) and stromal cells (dim signal). **c** Fractions of glioma cells associated with blood vessels, identified by vimentin staining with human-specific antibody. **d** Average distance of U-251 and E-98 cell migration in mouse brain slices. Values display median (*black line*), 25/75 percentiles (*boxes*) and maximum/minimum (*whiskers*) from three independent experiments. *Scale bars* 100 μm

The brain blood vessels have a complex anatomical and molecular organization (Yousif et al. 2013; Di Russo et al. 2017), and in vitro assays fail to reconstitute these microanatomic features. To recapitulate the perivascular niche for U-251 and E-98 cell invasion and validate the results obtained in rBM culture, we used organotypic brain slice culture (Fig. 4a). U-251 and E-98 cells both invaded the brain slice tissue effectively and preferentially associated with blood vessels (Fig. 4b, c). Using end-point analysis of the position of individual cells relative to the spheroid boundary, the invasion speed was ~10–50 µm/day (median ~ 25 µm/day) for both cell lines (Fig. 4d), similar to the invasion speed in 3-day matured astrocyte scaffolds (Fig. 3d). The invasion pattern of U-251 and E-98 glioma cells in brain slice culture was adaptive and was dominated by collective cell strands while extending along blood vessels (Fig. 4b, arrowheads) and occasional detached single cells (Fig. 4b, asterisks). Thus, glioma cell invasion from spheroid culture on brain slices displays plastic adaptation in dependence of the tissue subregion.

### Validation of in vitro assays by glioma invasion in vivo

**Fig. 5:**
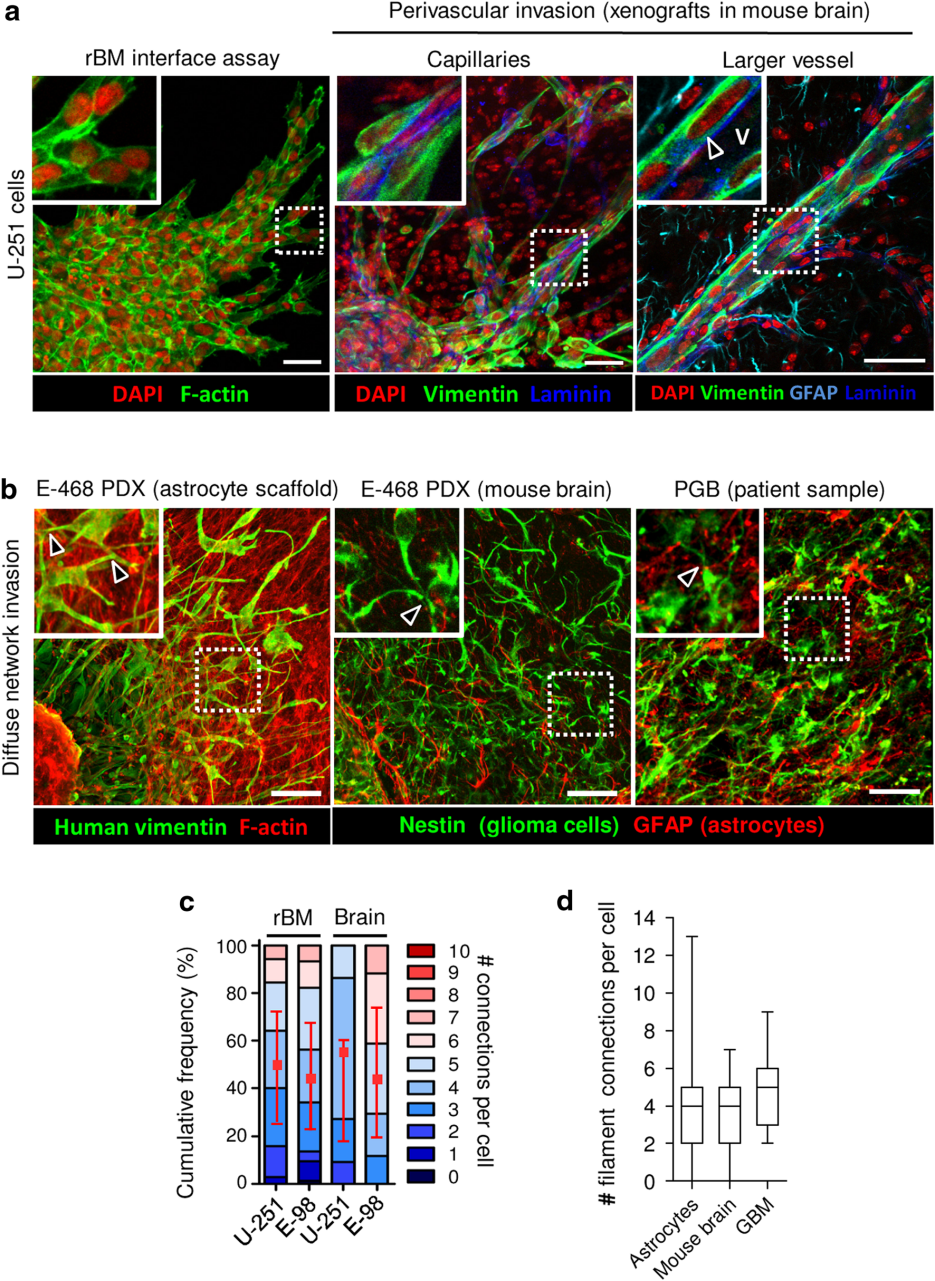
Validation of in vitro assays by glioma invasion in murine and human brain in vivo. **a** 3D reconstruction of U-251 cell invasion along rBM-plastic interface in vitro compared to invasion pattern in the mouse brain 1 month after orthotopic implantation of U-251 cells. Glioma cells were identified using human-specific anti-vimentin, basement membranes with anti-laminin, and astrocytes and glia limitans perivascularis with anti-GFAP antibody. *Arrowhead* indicates glioma cells invading along basement membrane of a linear brain vessel under glia limitans. V–vessel lumen. In vivo images are projections from 100 μm-thick z-stacks. **b** E-468 patient-derived glioblastoma cells invading (2 days) 3D astrocyte scaffolds in vitro, or mouse brain 2 months after orthotopic implantation, compared with the peritumoral region of a primary glioblastoma (PGB) patient sample. Images represent 100 μm-thick z-stacks. *Arrowheads* denote contacts between glioma cells via dendrite-like filaments. Glioma cells were positive for vimentin (E-468) or nestin (E-468 and human sample), detected with human specific antibodies. Astrocytes were detected by anti-GFAP antibody. **c** Number of cell–cell junctions between U-251 and E-98 glioma cells in different assays, including collective strands under rBM compared to perivascular invasion in mouse brain. Values represent the number of cell contacts per glioma cell (*colour code of stacked boxes*) and their relative frequency in the population. The number of connected neighbour cells is indicated as median (*red square*), 25/75 percentiles (*whiskers*), representing three independent in vitro experiments and 2 mice per cell line in mouse brain. **d** Number of filaments connecting glioblastoma cells during astrocyte scaffold invasion compared with mouse brain tissue and primary glioblastoma lesion. Values display median (*black line*), 25/75 percentiles (*boxes*) and maximum/minimum (*whiskers*). Data represent three independent in vitro experiments; two E-468 xenografts in mouse brain, and four glioblastoma patients. *Scale bars* 50 μm

To benchmark each in vitro invasion model, we compared the respective invasion patterns obtained in rBM, 3D astrocyte scaffolds and brain slice cultures with brain invasion in vivo, using 3D reconstructions of patient-derived xenografts in mouse brain and glioblastoma patient samples (Fig. 5a, b). Orthotopically injected in mouse brain, perivascular invasion of U-251 and E-98 glioma cells progressed as collective, finger-like strands along capillaries and larger blood vessels (Fig. 5a), and this pattern was reminiscent to their cohesive strand migration along rBM interfaces (Fig. 5a). Among other invasion patterns, similar cohesive, strand-like glioma cell invasion along blood vessels were previously observed by intravital two-photon microscopy in the mouse brain (Winkler et al. 2009; Watkins et al. 2014). The number of connections per cell in perivascular invasion strands was similar for in vitro rBM and in vivo mouse models, with 70% of the cells in direct contact with 3–7 neighbor cells (Fig. 5c). rBM is often used for coating transwell filters to model cell invasion through, rather than along, basement membrane (Benton et al. 2014). However, the data from the perivascular invasion in vivo confirm that glioma cells preferentially migrate along basement membranes and follow the perivascular space, but typically do not intravasate (Farin et al. 2006; Watkins et al. 2014).

Likewise, the in vitro/in vivo correlation was high for multicellular glioma network organization. Glioma cells connected with long filaments were found both in peritumoral regions of E-468 patient derived xenografts (PDX) in the mouse brain and glioblastoma lesions from patient samples (Fig. 5b, arrowheads), which was consistent with the filament-based network-like pattern of E-468 cells (Fig. 5b) during infiltration of 3D astrocyte scaffolds. The number of filament-based connections between E-468 cells in 3D astrocyte scaffolds (median four connections/cell) matched the number of filamentous connections between E-468 cells in the mouse brain (median four connections/cell) as well as in human glioblastoma samples (median five connections/cell) (Fig. 5b). The plasticity of glioma cell invasion modes, including cohesive strand- and sheet-like structures along rBM interfaces and in perivascular space, and diffuse multicellular networks in astrocyte scaffolds likely reflect the geometry of the environment, including microtracks of least resistance and confinement effects (Monzo et al. 2016).

Thus, the rBM interface and 3D astrocyte scaffold assays reliably represent two major routes of glioma dissemination in brain tissue in vivo: strand-like collective perivascular invasion along basement membranes and multicellular networks in astrocyte-rich stroma. Because rBM interface and astrocyte scaffold invasion assays are performed in 96-well plate format, both assays may be amenable for pharmacological compound screens and other experimental treatments.
